# New microsatellite markers for pigeonpea (*cajanus cajan *(L.) millsp.)

**DOI:** 10.1186/1756-0500-2-35

**Published:** 2009-03-06

**Authors:** DA Odeny, Jayashree B, C Gebhardt, J Crouch

**Affiliations:** 1 University of Bonn, Centre for Development Research (ZEFc), Walter-Flex Str.3 53113 Bonn, Germany; 2 International Crops Research Institute for the Semi-Arid Tropics (ICRISAT), Patancheru 502 324, Hyderabad, India; 3 Max-Planck Institute for Plant Breeding Research (MPIZ), 50829 Köln, Germany; 4 International Maize and Wheat Improvement Centre (CIMMYT), CP 56130, El Bat&#225n, Mexico

## Abstract

**Background:**

Pigeonpea is a nutritious tropical legume with several desirable characteristics but has been relatively neglected in terms of research. More efficient improvement can be achieved in this crop through molecular breeding but adequate molecular markers are lacking and no linkage map has been developed so far. Microsatellites remain the markers of choice due to their high polymorphism and their transferability from closely related genera. The overall objective of this study was to develop microsatellite markers from an enriched library of pigeonpea as well as testing the transferability of soybean microsatellites in pigeonpea.

**Results:**

Primers were designed for 113 pigeonpea genomic SSRs, 73 of which amplified interpretable bands. Thirty-five of the primers revealed polymorphism among 24 pigeonpea breeding lines. The number of alleles detected ranged from 2 to 6 with a total of 110 alleles and an average of 3.1 alleles per locus. GT/CA and GAA class of repeats were the most abundant di-nucleotide and tri-nucleotide repeats respectively. Additionally, 220 soybean primers were tested in pigeonpea, 39 of which amplified interpretable bands.

**Conclusion:**

Despite the observed morphological diversity, there is little genetic diversity within cultivated pigeonpea as revealed by the developed microsatellites. Although some of the tested soybean microsatellites may be transferable to pigeonpea, lack of useful polymorphism may hinder their full use. A robust set of markers will still have to be developed for pigeonpea genome if molecular breeding is to be achieved.

## Background

The increasing concern of the effect of global climate change and its likely impact on agriculture has stimulated scientists to search for crops that can withstand extreme environmental conditions. Among legumes, pigeonpea {*Cajanus cajan *(L.) Millspaugh} (2n = 22) has attracted attention as being both drought-tolerant [1] and highly nutritious [2]. Extensive morphological variation within the genus *Cajanus *as a whole and in cultivated species in particular has always led to the assumption that there exists abundant genetic diversity within the cultivated species. To the contrary, molecular studies have reported extremely low levels of polymorphism within the cultivated species compared to its wild relatives [3,4]. Such findings suggest that efforts towards the development of a linkage map of pigeonpea should focus on the use of an interspecific cross, and the development of a substantially high number of markers. We report the development of new 36 polymorphic simple sequence repeat (SSR) markers that will be an asset in characterising and understanding the nature of diversity within *Cajanus *species.

## Results

A total of 641 non-redundant contigs were generated from 2,131 sequenced clones reflecting an overall redundancy level of 70%. Of the 641 contigs, 117 sequences (20%) contained a microsatellite. The average size of each contig was 500 bp. This library thus covered an estimated 320,500 bp of pigeonpea genome. On the whole, di-nucleotide repeats were the longest (average 27 bp long) and also the most abundant followed closely by tri-nucleotide repeats (average 25 bp long). The longest motif was a 258 bp perfect hexa-nucleotide (AAACCC) repeat while a GT had the longest uninterrupted repeat of 74. A list of all designed pigeonpea primer sequences, SSR motifs and PCR programmes used for amplification is provided [see Additional file 1].

Table 1 gives a detailed characterisation of 35 pigeonpea primers that were polymorphic among the 24 diverse genotypes. Di-nucleotide repeats formed the highest proportion of polymorphic markers followed by tri-nucleotide repeats. The number of alleles detected ranged from 2 – 6 at each of the 35 polymorphic loci with a total of 110 alleles and an average of 3.1 alleles per locus. Gene diversity values ranged from 0.07–0.76 with an average of 0.41. While TG class of repeats formed the highest proportion (40%) of all the polymorphic loci, the highest number of alleles (6) was observed from a perfect tri-nucleotide repeat (CCttc019). The most informative marker with polymorphism information content (PIC) of 0.76 was a tri-nucleotide compound repeat (CCttc005), which was also the longest motif.

**Table 1:** PCR conditions, allele sizes and core motifs of new microsatellite loci that amplified in 24 different genotypes of pigeonpea

**SSR Name**	**Core Motif**	**Allele size range**	**No. of alleles**	**PIC^1^**
CCttc002	(gaa)5g(gaa)5	184–215	5	0.63
CCttc004	(gaa)6	240–250	2	0.08
CCac003	(ca)8	175–198	5	0.72
CCttc005	(gaa)11gag(gaa)5gaggaagag(gaa)17	305–320	5	0.76
CCac004	(ta)5(tg)7ta(tg)4	243–245	2	0.08
CCttc007	(aga)5	265–275	4	0.54
CCtc003	(tc)8	175–190	2	0.07
CCtc005	(ag)20	165–185	4	0.59
CCtta006	(att)21	290–310	5	0.71
CCcttc001	(cttc)4	260–270	4	0.28
CCgaaa001	(cttt)4	220–225	2	0.37
Cccta001	(gat)5(tct)(gat)4	275–302	3	0.29
CCac006	(ca)10cg(ca)6	295–350	5	0.49
CCgtt002	(tgt)4	210–235	3	0.40
CCttc012	(ttc)7	170–190	3	0.42
CCac007^1^	(tg)(tc)2(tg)7	275–280	2	0.08
CCgtt003	(ttg)5(ttc)7	165–180	2	0.37
CCtc007	(tc)6	310–330	4	0.68
Ccac010	(ca)7	191–198	3	0.50
Ccac011	(gt)7	227–270	2	0.36
CCttc017	(aga)11(ggag)(gaa)4ga(gga)3a(gaa)16	145–150	3	0.37
Ccat006	(ta)7(ca)6	220–277	3	0.54
Cccta003	(gat)4	420–450	3	0.39
CCac015	(ac)4aa(ac)38c(ca)7	135–145	2	0.14
CCtc009	(tc)6	200–220	4	0.45
CCac017^1^	caccac(a)5(ca)6c(a)4	215–227	2	0.24
CCac018	(ac)6a	200–210	3	0.54
CCac019	(tg)6	130–135	2	0.36
CCac026	(ac)7	278–295	4	0.64
CCac030	(cata)3ta(tg)6	236–244	2	0.37
CCttc018	(aga)5	275–288	2	0.35
CCac027	(tg)7	295–300	2	0.21
CCttc019	(aag)13	220–245	6	0.66
CCttc020	(ctt)8	236–242	2	0.37
CCac029	(caa)(ca)6caa	160–180	3	0.33

^1^Polymorphism Information Content

Optimum primer conditions were established for 39 (Table 2) out of the 220 (17.7%) soybean primers tested. One of the amplifying soybean primers revealed polymorphism among 24 diverse cultivated pigeonpea germplasm. The polymorphic microsatellite was an ATT-repeat (ATT_18_) and showed 4 alleles ranging from 290 to 335 bp. Twenty four of the amplifying soybean SSRs were genomic while the rest were EST-SSRs (Table 2). This means a higher proportion (25.4%) of the EST-SSRs designed could amplify pigeonpea DNA compared to 9.2% of the genomic SSRs tested. The EST-SSRs had relatively shorter motif lengths compared to the genomic ones. The common problems with amplification products for soybean SSRs were the appearance of excess number of bands, smears, and amplification failure.

**Table 2:** Characteristics of soybean microsatellite loci that amplified in pigeonpea.

**Locus^1^**	**Motif**	**Allele size/range**
Satt 173	(ATT)18	185
Satt 225	(ATT)13	150
Satt 239	(ATT)22	145
Satt 258	(ATT)9	115
Satt 259	(ATT)14	115
Satt 293	(AT)13(ATT)	170
Satt 308	(ATT)21	195
Satt 312	(ATT)11	250
Satt 328	(ATT)11	305
Satt 331	(ATT)14	150
Satt 336	(ATT)14	165
Satt 337	(ATT)19	255
Satt 339	(ATT)26	320
Satt 340	(ATT)12	230
Satt 343	(ATT)13	165
Satt 371	(ATT)11	390
Satt 403	(ATT)10	240
Satt 409	(ATT)27	315
Satt 442b	(ATT)35	400
Satt 541	(ATT)22	280
Satt 563b	(ATT)18	295–320
Satt 595	(ATT)9(GTT	445
Satt 599	(ATT)10	245
Satt 712	(ATT)21	350
SP 001	TGCTC(3)	175
SP 003	TGA(4)	500
SP 004	AT(26)	130
SP 010	CAA(6)	285
SP 028	TTA(4)	190
SP 032	CAA(5)	135
SP 040	CT(6)	400
SP 041	ATTC(5)	400
SP 047	TC(12)	145
SP 048	GAGAA(3)	270
SP 050	TATAT(3)	500
SP 053	CT(8)	300
SP 055	CCA(4)	230
SP 057	TA(10)	310
SP 059	CGCA(5)	200

^1^All loci with names starting with "SP" are EST-SSRs identified from the public database.

All markers shown were amplified in 24 genotypes of pigeonpea.

## Discussion

This study has resulted in the development of 112 new pigeonpea microsatellites; 73 *de novo *and 39 by transfer from soybean. The addition of these markers bring the numbers of microsatellite loci immediately available for testing in an interspecific cross to a total of 142. Due to the low genetic diversity within cultivated species, use of an interspecific cross would be the best strategy towards the development of a linkage map in pigeonpea. Even though wild relatives were not included in the current investigation, a past study [4] using microsatellites gives a good indication that these markers would be more informative among wild relatives than reported here for the cultivated species alone. Primers transferred from soybean will still have to be tested further to establish their informative nature for other species of the *Cajanus *genera. Such studies would be necessary to justify any future efforts towards the use of soybean primers in pigeonpea.

The average number of alleles per locus in this study was 3.14. Previous diversity analysis of cultivated pigeonpea species reported an average of 3.10 for 10 polymorphic loci [5] and 3.4 for 9 polymorphic loci [4], which are similar to the present results. This level of diversity is lower than 4.8 that has been reported when wild relatives are included in similar analysis [4]. Less diversity within cultivated pigeonpea has also been reported while using other markers [3]. Future breeding strategies in pigeonpea must focus on broadening genetic base by accumulating favourable alleles from other landraces and wild populations in order to maximise gains from selection. In tomato (*Lycopersicon esculentum*) for example, the development of an interspecific mapping population [6] greatly enhanced molecular breeding studies despite the low genetic diversity [7] reported.

## Conclusion

Pigeonpea germplasm collection at ICRISAT is already benefiting from the current study by utilising the markers developed to characterise a representative collection. These markers are expected to be beneficial in future for interspecific crosses and comparative genome analysis between the different *Cajanus *species for more efficient exploitation of the desirable characteristics therein. A larger number of markers would still be required in future to enable marker-assisted selection (MAS) in pigeonpea. With the current efforts to make DArT technology [8] available in pigeonpea [3] and the falling prices of DNA sequencing and SNP assays [9], more superior markers will undoubtedly be incorporated to complement the current efforts and enhance molecular marker technology in pigeonpea.

## Methods

### Development of an enriched genomic library

Genomic DNA was extracted from accession ICP 2376 and purified as described by Oberhagemann and colleagues [10] for the development of a small enriched genomic library. Ten μg DNA was digested with 50 enzyme units (*Sau*3AI) in a volume of 100 μl overnight at 37°C. The size of the DNA fragments was monitored by separating a 10 μl aliquot of the restricted DNA on a 1.5% agarose gel. The rest of the DNA fragments were ligated by T4 DNA ligase onto *Mlu*I self-complementary adaptors *RSA *21 and phosphorylated *RSA *25 according to Billotte and co-workers [11]. Twenty-five ng of the adaptor-ligated DNA was amplified through PCR in a 25-μl-reaction mixture that contained 1 μM *RSA *21, 2 μl of 2.5 mM dNTP, 1× PCR buffer (50 mM KCl, 10 mM Tris-HCl, pH 8.3), 1.5 mM MgCl_2 _and 2.5 units of *Taq *polymerase. The PCR program involved initial denaturation at 95°C for 1 min followed by 28 cycles of 94°C for 40 sec, 60°C for 1 min and 72°C for 2 min, and a final extension at 72°C for 5 min. Ten μl of each PCR product was viewed on a 1.2% agarose gel to confirm amplification. The rest of the PCR product was purified using QIAquick spin columns (Qiagen, Hilden, Germany) according to the manufacturer's instructions and resuspended in 100 μl of water. Microsatellite sequences were selected using biotinylated GA_8_, GT_8_, GAA_8 _and TAA_8 _oligonucleotide probes and streptavidin-coated magnetic beads following the hybridisation based capture methodology adapted from Billotte and co-workers [11].

The selected fragments were once more amplified by PCR (20 cycles), purified using the QIAquick columns (Qiagen) and concentrations adjusted to approximately 25 ng μl^-1 ^before cloning into pGEM-T vector (Promega, Madison, USA) according to the supplier's instructions. Positive colonies were handpicked and subjected to colony PCR using T7 and SP6 primers (Metabion, Martinsried, Germany). All products with an insert size range of 300 – 1100 bp were purified for sequencing using EXOSAP (Amersham Biosciences, Freiburg, Germany). Sequencing, sequence analysis and primer design was done as described elsewhere [4]. Primers were designed for 113 loci of which 73 [see Additional file 1] amplified single discrete bands.

### Testing of soybean (*Glycine max *L.) microsatellites for amplification in pigeonpea

Additionally, 161 genomic soybean SSRs (100 of which were kindly provided by Dr. Perry Cregan of the United States Department of Agriculture) that were previously mapped in soybean [12] and 59 soybean EST (Expressed Sequence Tags)-SSRs were tested for amplification in pigeonpea using soybean cultivar *Williams*' DNA as a control. More details on the primer sequences and Genbank accession numbers of the EST sequences are also available [see Additional file 2].

### Testing amplifying loci for polymorphism in pigeonpea

The amplifying loci of both soybean and pigeonpea primers were tested for polymorphism among 24 diverse pigeonpea accessions (ICP 13575, ICP 15145, ICP 9266, ICP 4167, ICP 14576, ICP 12058, ICP 14352, ICP 1514, ICP 7543, ICP 7852, ICPL 87091, ICP 7035, ICPL 151, Kat 60/8, HPL 24, LD Dwarf, ICPL 99066, MN 5, ICPL 332, ICPA 2068, ICPA 2032, ICP 13092, ICPL 87119, ICP 2376). All seeds were obtained from ICRISAT, India. PCR reactions were prepared in 10 μl volumes and amplification was confirmed in 1.2% agarose gels by loading 5 μl PCR products of randomly selected samples. Amplification products were thereafter visualised on non-denaturing 6% 29:1 (w/w) acrylamide/bisacrylamide gels followed by silver staining. The bands visualized on the gels were scored using a binary code for presence ("1") or absence ("0") of bands (alleles) for every SSR locus. Respective allele sizes were estimated using various commercial DNA ladders (Bioline, UK). The polymorphism Information Content (PIC) was calculated as described by Botstein and co-workers [13] using the formula below;

$$PIC=1-\left[\sum_{i=1}^{n}p_{i}^{2}\right]-\left[\sum_{i=1}^{n-1}\sum_{j=i+1}^{n}2p_{i}^{2}p_{j}^{2}\right]$$

where *p*_*i *_equals the frequency of the *i*th allele and *p*_*j *_the frequency of the (*I *+ 1)^th ^allele. Only data from polymorphic SSR loci were used for this analysis.

## Competing interests

The authors declare that they have no competing interests.

## Authors' contributions

DO carried out the data collection, data analysis and drafted the manuscript. JB participated in sequence analysis, soybean marker mining, primer design, and editing of manuscript. CG supervised the project, critically reviewed and commented on the manuscript. JC conceived of the study, participated in the overall design and implementation of the project. All authors read and approved the manuscript.

## Supplementary Material

Additional file 1 **Pigeonpea SSR motifs, primer sequences and PCR amplification conditions.** Click here for file

Additional file 2 **Soybean EST-SSR primer sequences and amplification conditions.** Click here for file
