# Supplementary material for: New microsatellite markers for pigeonpea (cajanus cajan (L.) millsp.)
Source: BMC Res Notes. 2009 Mar 6;2:35. doi: 10.1186/1756-0500-2-35 (PMC2660351; doi:10.1186/1756-0500-2-35)
Supplement: Additional file 1 — Pigeonpea SSR motifs, primer sequences and PCR amplification conditions. [file 1756-0500-2-35-S1.doc]

**Additional File 1**

**Pigeonpea SSR motifs, primer sequences and PCR amplification conditions**

| **Accession number** | **Motif** | **Primer Sequences** | | **Amplification** | **Touchdown^1^ (^0^C)** | |
| --- | --- | --- | --- | --- | --- | --- |
| CZ681920 | ca8 | F: gcgggattctcttgcttac  R: tcacaaaacaatttggcaca | | Yes | 55-45 | |
| CZ681921 | tct6 | F: gggaattttgttggggtttt  R: tgcttacgcgtggactaatg | | No |  | |
| CZ681922 | (gaa)5g(gaa)5 | F: acaccaccatgctaaagaacaag  R: ccaagcaagacacgagtaatcata | | Yes | 60-55 | |
| CZ681923 | aag5 | F: catcgcctacaatcatacaaaga  R: tcttgtcctttttcagtcatcgt | | Yes | 60-55 | |
| CZ681924 | gaa6 | F: atcgctttgcatccttatc  R: cttcacgtacattttcgttt | | Yes | 60-55 | |
| CZ681925 | ca8 | F: tgcttcaagttgcctaccag  R: tcaagggaggtggactacaaa | | Yes | 60-55 | |
| CZ681926 | (gaa)11gag(gaa)5gaggaagag(gaa)17C | F: gtagaggaggttccaaatgacata  R: atctgtctggtgttttagtgtgct | | Yes | 60-55 | |
| CZ681927 | gaa16 | F: ctcttgcttacgcgtggact  R: cttttgcttttgcgtgctt | | Yes | 55-45 | |
| CZ681928 | (ta)5(tg)7ta(tg)4 | F: tcttagcatgtcctctattttcgt  R: agtacatttcaaatccacacatcc | | Yes | 60-55 | |
| CZ681929 | aga5 | F: tcacagaggaccacacgaag  R: tggactagacattgcgtgaag | | Yes | 60-55 | |
| CZ681930 | tc8 | F: gcgctaagggaaaacaaaaa  R: aactcccttgttgtcatatggtg | | Yes | 60-55 | |
| CZ681931 | ca9 | F: cgtctatggagggttttcag  R: agacatttatcaatccaaggtg | | No |  | |
| CZ681932 | ttc9 | F: ttcttcttcttcttctcctttcttc  R: ccatcatcttcaactgcgata | | No |  | |
| CZ681933 | aga4 | F: agagggaaagggaagagaaga  R: tcaagcaactccaagaaattca | | Yes | 60-55 | |
| CZ681934 | cttc(ctt)4 | F: aaggcttttcaacaaataggg  R: agaagagaaaaagcataaaacttca | | Yes | 60-55 | |
| CZ681935 | tc8 | F: catttatttctctctggcattcac  R: cgagctgcaagcataaacg | | Yes | 60-55 | |
| CZ681936 | ac53 | F: gccattacttgagtgtgagttttg  R: gtgtgtgtgtgtgtgtgtgtgtgt | | No |  | |
| CZ681937 | ag20 | F: tgcacagattcgaaggttcc  R: cctcaagattcctctttctctca | | Yes | 60-55 | |
| CZ681938 | att21 | F: tcaggggtaaatgcggtatc  R: gaattgctttttgcttcctca | | Yes | 65-60 | |
| CZ681939 | gggaga4 | F: gagaaatatgagaggcagagagaga  R: aagataattcattagggggtgga | | Yes | 60-55 | |
| CZ681940 | cttc4 | F: taaggaaatggctggggttg  R: cacataaatttgggggttcg | | Yes | 55-45 | |
| CZ681941 | cttt4 | F: ggacttgttactggggcact  R: aattcccatggtcattcg | | Yes | 55-45 | |
| CZ681942 | gac4 | F: cctatcggaaggagaaaaacatt  R: tcgctaaagtcttggtagataatgg | | No |  | |
| CZ681943 | (gat)5(tct)(gat)4 | F: tgggcatggtagaggaagtt  R: cgtcatgaagcaacaggaga | | Yes | 55-45 | |
| CZ681944 | ga9 | F: cgtgaatggctgactctatgaa  R: tcttgagcttgcatcctcct | | No |  | |
| **Accession number** | **Motif** | **Primer Sequences** | | **Amplification** | **Touchdown^1^ (^0^C)** | |
| CZ681945 | aaaccc43 | F: ccggtttagggtttagggttt  R: ggttggagggtttagggttg | | No |  | |
| CZ681946 | ctt4 | F: taatcccattccgttgtcgt  R: cccaggaagagatgagacca | | Yes | 60-55 | |
| CZ681947 | ca7 | F: aggctttctcccttcaatcc  R: gccttttcaaacttttctcaca | | Yes | 60-55 | |
| CZ681948 | tttc4 | F: tcttcatcctcactcttccctaa  R: gaggtgcccaaggaagatag | | No |  | |
| CZ681949 | tc13 | F: ccttttctttgtcggaatcactaa  R: cggaggctgttggatctagtattt | | No |  | |
| CZ681950 | agg4 | F: ggggtgaatggtagtggaaa  R: tccctctctcctccccttat | | No |  | |
| CZ681951 | (ca)10cg(ca)6 | F: acatgtgtggcgtagtgtga  R: gcaaaaccgttccataaaaa | | Yes | 65-60 | |
| CZ681952 | tct29 | F: ccagccggatcgttacacta  R: tggtagattttctcgtgactgc | | No |  | |
| CZ681952 | ata4 | F: gcagtcacgagaaaatctaccac  R: ggttgattatcgaatgaaatggag | | No |  | |
| CZ681953 | gaa47 | F: tgggcatggtagaggaagtt  R: catcataatcgtcttcatcacttg | | No |  | |
| CZ681954 | tc7 | F: aaaaatttcgtccaaagctcct  R: ggaagattgaattacatacctctcg | | No |  | |
| CZ681954 | tc7 | F: gaggattgcaccaagcaact  R: gcactgctggccttaccata | | Yes | 55-45 | |
| CZ681955 | tgt4 | F: tgggctgtgatcgatgaat  R: cgacaacaacaacaccgact | | Yes | 60-55 | |
| CZ681956 | tgc)4(tga)2 | F: cgggattctccttgccttac  R: gcagcatcatcatcactacga | | No |  | |
| CZ681957 | aga4 | F: tgttccgtttcaagtggtca  R: cgacatttacccactcgttca | | Yes | 55-45 | |
| CZ681958 | ttc7 | F: tagagcgttgtcccttttctg  R: tcgaaggacaactcaagcatt | | Yes | 65-60 | |
| CZ681959 | aga9gaaagaa | F: tgggcatggtagaggaagtt  R: cccaccattaccaagcaagt | | No |  | |
| CZ681960 | at6 | F: tcgtgggaatgctctacaac  R: aaccacaagtacacccacacc | | Yes | 65-60 | |
| CZ681961 | aga10 | F: atgggcatggtagaggaggt  R: cgctcatcatcgtcatcaaa | | Yes | 55-45 | |
| CZ681962 | (tg)(tc)2(tg)7 | F: gggaaactcacctatattaccaa  R: cactaccgtctacagccatctc | | Yes | 65-60 | |
| CZ681963 | (ttg)5(ttc)7 | F: gttcttcttgttgttgttgttg  R: aattcgtggagttcattgg | | Yes | 65-60 | |
| CZ681964 | (ca)7aca(ta)3 | F: gatagcacacacacacacaaca  R: taccttagggtcaccaacga | | Yes | 65-60 | |
| CZ681965 | gaaaaa5 | F: ctttgttcagagcggagcat  R: tttttaggacattgggaagca | | Yes | 65-60 | |
| CZ681966 | (ttc)4tgc(ttc)3 | F: agtcgatgtggaacatgagga  R: tgttgtaagccgtgggtagg | | Yes | 55-45 | |
| CZ681967 | (gaa)2gagg(gaa)4gag(gaa)2 | F: aggtgcaaaggaagcactaat  R: cagctccactgtcttcaacg | | Yes | 60-55 | |
| CZ681968 | att4 | F: caggattttaatggattctgcaa  R: gggtgaatactatttaaaaggatagg | | Yes | 65-60 | |
| **Accession number** | **Motif** | **Primer Sequences** | | **Amplification** | **Touchdown^1^ (^0^C)** | |
| CZ681969 | act4 | F: atcccagacttcatagggagatag  R: gtctagtcccaggtacaaagaggt | | Yes | 60-55 | |
| CZ681970 | tc6 | F: cttctccctgcctcttttcc  R: caagtggaggggagtgaaga | | Yes | 55-45 | |
| CZ681970 | ctt4 | F: tcctctctcctcttgtctttgtc  R: atggagaagtgaaagggatatgt | | No |  | |
| CZ681971 | ca8 | F: aagttgcctactgggggttc  R: aaatagagctgtcaggggaggt | | Yes | 65-60 | |
| CZ681972 | att5 | F: tgcatgatatgagatgatggaga  R: cccttttcacccaaaaatacaa | | No |  | |
| CZ681973 | gaa6 | F: tggactaccaaacgcagaca  R: tcgtagctgcagagcatttt | | Yes | 65-60 | |
| CZ681974 | ctc4 | F: atcctccaaaagttccacca  R: caaaggaggatttccaccaa | | Yes | 55-45 | |
| CZ681974 | tct4 | F: cggccccttctatactgtca  R: gaaaagagaaaagaaggaaagagga | | No |  | |
| CZ681975 | cat6 | F: acggtgccttgttgattgta  R: cggaacaggaggaaaaggtc | | Yes | 55-45 | |
| CZ681976 | ag)5aac(ga)4 | F: gcgaagagggtaaagggaaa  R: ccggtcacgagaaatgtgta | | No |  | |
| CZ681977 | ca7 | F: accttgcttgtttcgctttt  R: aagggaggtggactacaagga | | Yes | 65-60 | |
| CZ681978 | tc9 | F: tgcaagcttgtattctatagtgtc  R: atggagatttaggtgctttgtg | | No |  | |
| CZ681979 | gt7 | F: gtgagtgagagtgagtgtatttgtg  R: gctctgatgccaaatgttga | | Yes | 65-60 | |
| CZ681980 | ttc5 | F: catccattgggttgttctca  R: ggattaaagcgcaccatcat | | No |  | |
| CZ681981 | (tg)6cgagtgtga(gt)11gcaacta(tg)6 | F: catcataatcatacatgtcaatgcta  R: ggttttatctttgtctccaattctg | | Yes | 55-45 | |
| CZ681982 | ac)10c(ca)74 | F: tgggaaacaaaatatcccctaa  R: agaggggtgtgatgaagcag | | No |  | |
| CZ681983 | (aga)11(ggag)(gaa)4ga(gga)3a(gaa)16 | F: tgggcatggtagaggaagtt  R: tcagaagtcgatggcaagtg | | Yes | 55-45 | |
| CZ681983 | tga11 | F: gaggaggaggaagaagaagaaga  R: tcgtcgccgtatcactacaa | | No |  | |
| CZ681984 | ac53 | F: catgcgtattgaatgaattg  R: tctcgtctgagtgggagtgt | | No |  | |
| CZ681985 | gt8 | F: gcccctcttacaccttttctt  R: ctcttgcttacgcgtggact | | No |  | |
| CZ681986 | (ta)7(ca)6 | F: tgctctaatggctagttcatcc  R: aaacactcatgggttagattctcc | | Yes | 65-60 | |
| CZ681987 | ttc8 | F: tcttgcttacgcgtggacta  R: tggagaagggacacaaatgc | | No |  | |
| CZ681988 | ttccc)(ttc)3tcc(ttc)4taca(tct)7 | F: ttacctgacgtgaagtgaatgg  R: cgtgcgacaggactacaatg | | No |  | |
| CZ681989 | gat4 | F: tagtatgggcgtggtagagga  R: cgtgacagagtcaatcagaagc | | Yes | 55-45 | |
| CZ681990 | (tg)6(agtg)3 | F: caggtctgctactgccatca  R: agcccacttctgcatcactc | | Yes | 60-55 | |
| CZ681991 | (ac)7(ca)3 | F: ccacatccctcaacccatac  R: gaaaagcccttgatgacacc | | Yes | 60-55 | |
| **Accession number** | **Motif** | **Primer Sequences** | | **Amplification** | **Touchdown^1^ (^0^C)** | |
| CZ681992 | ttc4 | F: ttgtccgtagctctcgtttct  R: gctatgcagcggtaagtgtg | | No |  | |
| CZ681993 | (ga)4ca(ga)4cagagt(ga)8 | F: atcatcagattcttcagccgta  R: ggttagaccaatccaatcaagc | | Yes | 60-55 | |
| CZ681994 | (ac)4aa(ac)38c(ca)7 | F: gggaaacaaaatatcccctaatc  R: taatcacacacatcacacctagca | | Yes | 55-45 | |
| CZ681995 | (ac)6aag(ctaa)3 | F: cacgattccattggtggag  R: acggtttctgggagggtcta | | Yes | 60-55 | |
| CZ681996 | at6 | F: ccacaagtacacccacacca  R: ttcgtgggaatgctctacaa | | Yes | 60-55 | |
| CZ681997 | ag)6g(c)9 | F: ttgggaaatgaaggttgagc  R: gcgtggagtaatccatgaaaa | | No |  | |
| CZ681998 | tc6 | F: acaaatccggtgacccataa  R: ccgagaacaaaaacattgaaca | | Yes | 65-60 | |
| CZ681999 | caccac(a)5(ca)6c(a)4 | F: gactagaaaattcacctccgtctg  R: ttacaaaggctacattgatgagaac | | Yes | 65-60 | |
| CZ682000 | tg7a(gt)23 | F: ttgcttacgcgtggactaga  R: aacagtgggtgcatatgatttt | | No |  | |
| CZ682001 | ac6a | F: tctttcagacgcaatgacctt  R: cacttatttgtggggaccatc | | Yes | 60-55 | |
| CZ682002 | tg6 | F: caaggaatcacttaaaaaccaagc  R: agatggccaagattccacaac | | Yes | 60-55 | |
| CZ682003 | ca6(ta)6(ca)3 | F: catcaggcgttaggaactctc  R: ttgtggattgtgttatgtgtgc | | No |  | |
| CZ682004 | gt6 | F: gccttttcaaacttttctca  R: catatgctttaagtgctttcct | | Yes | 60-55 | |
| CZ682005 | ac6 | F: tgtatgttcgtttagaggcttcc  R: gccccttttcacttttctca | | Yes | 65-60 | |
| CZ682006 | ca6c | F: tgcctactaggggtttcgtg  R: tgaactatccagggaggtgag | | Yes | 65-60 | |
| CZ682007 | tgt)(ttg)2(tg)7 | F: tgatttgtgcttgtgccttg  R: gtcttgcttacgcgtggact | | No |  | |
| CZ682008 | ca8 | F: aacgatgaaattcccaaacg  R: tgttagatgctcaacccaagg | | Yes | 60-55 | |
| CZ682009 | tg7 | F: agccacttaataaccaagcctttt  R: gtgtatgctttacttgctttccttt | | Yes | 65-60 | |
| CZ682010 | aga6 | F: tcttcgctttgaggggacta  R: gggaattttgttggggtttt | | No |  | |
| CZ682011 | gt7 | F: aaattcaccaccatgatccaa  R: tcttcacttccgagacacaact | | Yes | 55-45 | |
| CZ682012 | caa)g(ca)5cg(ca)(ta)2 | F: tcaacacctgattaagatttgttcc  R: agggtttctcaagtggtaaggttt | | No |  | |
| CZ682012 | atg4 | F: caagaaagcacccctcgtag  R: ataggagcatccgtcgacaa | | No |  | |
| CZ682013 | ac7 | F: tgagaggcaatgatgttgga  R: tctacaggcaccctttgaaaat | | Yes | 60-55 | |
| CZ682014 | (cata)3ta(tg)6 | F: atcggcttttgtcttgatga  R: aagctacaagggatacacatgc | | Yes | 60-55 | |
| CZ682014 | ac8(at)7acat | F: ggccaagtcactgtcgaatc  R: tgtagtccacgcgtaagcaa | | No |  | |
| CZ682015 | aga5 | F: acaattactcaaatgctctcaacg  R: taaatgtcgcttcctatgatagacc | | Yes | 60-55 | |
| **Accession number** | **Motif** | **Primer Sequences** | **Amplification** | | | **Touchdown^1^ (^0^C)** |
| CZ682016 | tg7 | F: gacgtggtcattgaaagtagca  R: agacaaaaactacacgcactcaag | | Yes | 60-55 | |
| CZ682017 | aag13 | F: tgaaatgaacaaacctcaatgg  R: tgtattgcacattgacttggcta | Yes | | | 60-55 |
| CZ682018 | tct9 | F: ttggtcacatagttgtagagtgttg  R: aaagattactctgttgtcgtggat | No | | |  |
| CZ682019 | aat4 | F: aacacgcacctcaattcca  R: gaatgaggaatgaagggacaaa | Yes | | | 60-55 |
| CZ682019 | ctt8 | F: attccctctctatctcagactttt  R: tcgtgatggaactcaagatacact | Yes | | | 60-55 |
| CZ682020 | ag8 | F: gcggtgaagatggatggat  R: ctcttgcttacgcgtggact | No | | |  |
| CZ682021 | tg8 | F: ttagggtcaccagtgatgatatgt  R: tttcaggtgcagaaataaaggttag | Yes | | | 65-60 |
| CZ682021 | (caa)(ca)6caa | F: cgtggactaatcatcccgtaa  R: ataatgccaaagggggagaa | Yes | | | 60-55 |
| CZ682022 | aac4 | F: atcccgtaatgcaccttttg  R: ttggtctgaattgtggcctat | No | | |  |
| CZ682023 | tc13 | F: ctaggccctcgagctacatt  R: tcttttagaggtgcgctgtg | Yes | | | 55-45 |

*^1^See Odeny et al. (2007) for details on “Touchdown” PCR amplification*
