# Supplementary material for: New microsatellite markers for pigeonpea (cajanus cajan (L.) millsp.)
Source: BMC Res Notes. 2009 Mar 6;2:35. doi: 10.1186/1756-0500-2-35 (PMC2660351; doi:10.1186/1756-0500-2-35)
Supplement: Additional file 2 — Soybean EST-SSR primer sequences and amplification conditions. [file 1756-0500-2-35-S2.doc]

**Additional File 2**

**Soybean EST-SSR primer sequences, motifs** and amplification conditions

| **Gene Identifier** | **Locus name** | **Motif** | **Primer Sequences** | **Amplification^1^** |
| --- | --- | --- | --- | --- |
| 5058094 | SP 001 | TGCTC(3) | F: attgtttgattccgcttggt  R: ccgagagctaagaagccaac | Yes |
| 10847563 | SP 002 | TC(6) | F: cgattgggtgtaatggttgtt  R: ctgccttcaccattccagtt | No |
| 10843581 | SP 003 | TGA(4) | F: gggatcttctccctccaaag  R: aatggtagggggtgtccact | Yes |
| 7139589 | SP 004 | AT(26) | F: gattcttctgcctcgattgc  R: gacagcgtatgcctgaacaa | Yes |
| 9902130 | SP 005 | CTT(4) | F: ccgttaccggttattttgga  R: acatggcatggcatacataca | No |
| 9902130 | SP 006 | ATC(5) | F: agccatgagcgctctcttta  R: gggcaagggtgaaagaaaa | No |
| 8670030 | SP 007 | CAA(7) | F: ctaaggtccgttgccacaa  R: actgcttcgactccttgcat | No |
| 21994952 | SP 008 | TC(5) | F: gtgcaaagcatccttcgttt  R: tggtcttaccagcagcatca | No |
| 7795152 | SP 009 | TTCT(3) | F: cttgcaaatttttgcctggt  R: tggttaaaagcatgtgtgaactg | No |
| 4396518 | SP 010 | CAA(6) | F: atcatcagcagcagcaagaa  R: ttgttgttgaggaggtgtgg | Yes |
| 16996268 | SP 011 | AG(5) | F: agtaatttcggcggtgagaa  R: ccctgctctctcttctgcat | No |
| 15811822 | SP 012 | TTA(7) | F: ccaagggattcgtctgctac  R: tgtgagggaggctacaagaaa | No |
| 15425630 | SP 013 | GAA(5) | F: caggaaaagcaccaaggaaa  R: gaacgtggccatattggttt | No |
| 15243080 | SP 014 | TAT(16) | F: tttggacagaatggagaaagaaa  R: gggggatcaaaacatcacac | No |
| 14970126 | SP 015 | ATT(15) | F: tctgctaagggcaattttattca  R: ccagcagcctgttcagtttt | No |
| 14205849 | SP 016 | ATG(6) | F: tggaagaggatggaaccttg  R: gatcatttgcaagcggattt | No |
| 13396954 | SP 017 | TC(10) | F: gcgctgtcattgtgtctttg  R: ccccgtgatctaatcgagaa | No |
| 10254380 | SP 018 | AAT(5) | F: gacaaccaaaccacacacga  R: atccaccgagaagctgaaga | No |
| 19346726 | SP 019 | TC(19) | F: ccatagtagcgagggctgtg  R: accaaagcagtggggttaca | No |
| 19345944 | SP 020 | ATT(7) | F: cccatatttcgaagcccaat  R: tttttctggcgaaaggctta | No |
| 18039805 | SP 021 | GAA(4) | F: aacgcagcgataatcagagc  R: tggttttgctgctttaccaa | No |
| 17962853 | SP 022 | TA(5) | F: ggtgcctaatgagcctctgt  R: cgagtcaatttgatgccattt | No |
| 22521799 | SP 023 | AG(10) | F: cagtctcgactcaacggtca  R: ccattgtgttgcctgatcc | No |
| 6667821 | SP 024 | AAT(5) | F: gaaccaaccagagcacccta  R: tgccttatgtggaaagaacttg | No |
| 6454293 | SP 025 | TC(6) | F: cgcgtcttcattcctctcat  R: gaccacctcgatctcaggac | No |
| 7478267 | SP 026 | CTTC(4) | F: tccctcacaatcacaatgct  R: tgagcaatgaaaggtggttg | No |
| **Gene Identifier** | **Locus name** | **Motif** | **Primer Sequences** | **Amplification^1^** |
| 15203924 | SP 027 | GCGAG(3) | F: agagagagagagagagagaaaacagc  R: tcaggccttgatgtgatgtc | No |
| 7029704 | SP 028 | TTA(4) | F: tctccttcatcttggaagtgc  R: caaaacagtagggaagcaaaca | Yes |
| 22639035 | SP 029 | AAAT(3) | F: tggtgtctttggcaatctttt  R: gggtctattcattgtgagtttcg | No |
| 6747342 | SP 030 | GAT(4) | F: ggagccattgtgacagtgaa  R: accaatgttttggcgaagtc | No |
| 6747670 | SP 031 | CCT(6) | F: cggggaatttagtttttggag  R: gctgctgctgttgttgttgt | No |
| 6747670 | SP 032 | CAA(5) | F: acacactccctcctcctcct  R: tgttgttgctgctgttgttg | Yes |
| 6665892 | SP 033 | GAA(5) | F: ttgtccagtgctgagaaagc  R: tcatcatctccatcgtcttcttt | No |
| 22933319 | SP 034 | AATA(3) | F: ccacgtgcaccttctcttaaa  R: cgttgggctgctttacaaat | No |
| 22932563 | SP 035 | TG(7) | F: aaggcaagtcagccagaaaa  R: cttgccaatgcttctgagc | No |
| 23064154 | SP 036 | AT(5) | F: cgctaatggggtcatcaact  R: tgatggttttggcgagtaaa | No |
| 22931844 | SP 037 | AC(9) | F: atacaccttggccactttgc  R: cccagcttggcagatacatt | No |
| 22931676 | SP 038 | TCA(5) | F: tcctcctccccaagtaaagg  R: cctaccatcgtaccccattg | No |
| 22930793 | SP 039 | AAATC(4) | F: tagtgatcggcatggattga  R: gtggctgatgtagcccttct | No |
| 15815753 | SP 040 | CT(6) | F: ttcattgcatcaccgtcact  R: ctcctccttccattgctctg | Yes |
| 15815753 | SP 041 | ATTC(5) | F: ttcattgcatcaccgtcact  R: ctcctccttccattgctctg | Yes |
| 15664768 | SP 042 | CCA(4) | F: tttcaccaccctctgagctt  R: agcacttcaacagcagcaag | No |
| 15662703 | SP 043 | CAG(4) | F: tgtctgggattttgctctgtt  R: cgattcgagcgagctaagtaa | No |
| 15662703 | SP 044 | TTTGC(3) | F: tgtctgggattttgctctgtt  R: cgattcgagcgagctaagtaa | No |
| 23731669 | SP 045 | TTA(25) | F: caagaggttgcgacaattcc  R: tcactcaacaacaaaacaacaattt | No |
| 15200619 | SP 046 | CAG(4) | F: aatgcaggatccatttgctc  R: aaagggattgctgggttcat | No |
| 14125154 | SP 047 | TC(12) | F: ttgtgaccacaagccttcac  R: cgaggaaggtgacgtaggc | Yes |
| 13563100 | SP 048 | GAGAA(3) | F: gaggcggagacgaagaagt  R: ttgtagacgaggacgggaat | Yes |
| 29842360 | SP 049 | TTTTC(3) | F: ccaaatcaaaagtggcgtct  R: aggtgggtgggttttgtatct | No |
| 29844449 | SP 050 | TATAT(3) | F: tgtaccaaaccgtgaaaacg  R: ccatgcttctccaggttcat | Yes |
| 27809239 | SP 051 | AGATT(3) | F: ccttgaagcatcccaatatca  R: gccactggagcttttcgtta | No |
| 27425640 | SP 052 | TCC(4) | F: gattgccgagattcaaccat  R: atttccgacttggaggatga | No |
| 26057188 | SP 053 | CT(8) | F: atccctctcccctctctcac  R: tcctttgggttttggtcttg | Yes |
| **Gene Identifier** | **Locus name** | **Motif** | **Primer Sequences** | **Amplification^1^** |
| 26057188 | SP 054 | GAA(5) | F: catagtcaccgaccatgacg  R: ttgttccgatgaagtgcaag | No |
| 26048070 | SP 055 | CCA(4) | F: tcgcagtcatggtcaaagaa  R: tctaactcctcgccatgctt | Yes |
| 26047854 | SP 056 | TA(7) | F: tgcttctgtggctacctcaa  R: tgaatgcttgtgtcaagatgttt | No |
| 26045009 | SP 057 | TA(10) | F: caagcagagatggcccttag  R: gaagcgaccaattaccaaaca | Yes |
| 25898746 | SP 058 | CAA(4) | F: acgccgtgacccaaataata  R: tcatcaccacccatgttttg | No |
| 24136689 | SP 059 | CGCA(5) | F: cttcactttccccaactcca  R: cgttgtgatcactcgcagac | Yes |

*^1^All primers were amplified under “Touchdown” PCR with temperature range of 60-55^0^C*
